# The Relationship of School Start Times, Sleep Duration and Mental Health among a Representative Sample of High School Students in Colorado, 2019

**DOI:** 10.3390/ijerph18115708

**Published:** 2021-05-26

**Authors:** Lucas M. Neuroth, Ming Ma, Ashley Brooks-Russell, Motao Zhu

**Affiliations:** 1 The Center for Injury Research and Policy, Abigail Wexner Research Institute at Nationwide Children’s Hospital, Columbus, OH 43205, USA; Lucas.Neuroth@NationwideChildrens.org; 2 Department of Community and Behavioral Health, Colorado School of Public Health, University of Colorado Anschutz Medical Campus, Aurora, CO 80045, USA; Ming.Ma@CUAnschutz.edu (M.M.); Ashley.Brooks-Russell@CUAnschutz.edu (A.B.-R.); 3 Division of Epidemiology, College of Public Health, The Ohio State University, Columbus, OH 43210, USA; 4 Department of Pediatrics, College of Medicine, The Ohio State University, Columbus, OH 43205, USA

**Keywords:** sleep, school start times, adolescent, high school, suicide, mental health

## Abstract

This cross-sectional study utilized responses from 46,537 students enrolled in grades 9 through 12 in 166 high schools across the state of Colorado via the 2019 Healthy Kids Colorado Survey to: (1) quantify the association between high school start times and student sleep duration and (2) investigate the associations between school start times and student mental health. Descriptive and bivariate analyses were used to investigate associations between school start times and self-reported demographic, sleep, and mental health factors. Survey-weighted multivariate regression modeling was used to investigate associations between school start times, sleep duration, and mental health. Schools with late start times (≥8:30 a.m.) saw 32.2% (95% Confidence Interval: 29.5–35.0) of students sleeping 8 h or more relative to 23.2% (22.0–24.4) in schools with very early start times (<8:00 a.m.). For every 15 min later school start time, students’ sleep duration was 4.6 (3.4–5.9) min longer. Students attending schools with very early start times had 1.10 (0.95–1.27) times the odds of attempting suicide compared to those attending schools with later start times, while students at schools with early starts (8:00–8:29 a.m.) were associated with 1.11 (0.98–1.27) times the odds. Schools with later school start times had a statistically significantly higher proportion of students sleeping 8+ hours. Schools with start times before 8:30 a.m. had 10–11% higher odds of students attempting suicide compared to schools with late start times, though these differences were not statistically significant. Student mental health should continue to be investigated when assessing the potential impacts of delayed school start times.

## 1. Introduction

Globally, the proportion of adolescents achieving adequate sleep is cause for concern. While over 60% of adolescents are achieving adequate sleep in countries such as Sweden, Canada, Norway, and Belgium, only 30–40% of adolescents reach this amount of sleep in Poland, Greece, and Estonia [1]. Within the United States, the proportion of adolescents achieving adequate sleep, defined as 8 to 10 h per night for 13- to 18-year-olds by the American Academy of Sleep Medicine [2], is even lower. Recent data from the 2019 National Youth Risk Behavior Survey (YRBS) found only 22% of adolescents attending high school are sleeping 8 h or more on an average school night [3]. Further, there are disparities in sleep duration across grade levels, with high school seniors (12th graders) having the lowest proportion of adequate sleepers and freshman (9th graders) having the highest [4].

These findings are concerning when considering the numerous negative health outcomes, both physical and mental, that are associated with inadequate sleep in adolescents. In their 2014 systematic review, Shochat, et al. [5] highlight studies linking inadequate sleep to increased risk of mental and emotional disfunction, depressive symptomology, poor academic performance, and a wide variety of risky behaviors. Additionally, sleep duration has a curvilinear dose–response relationship with both suicidal ideation and attempts, with the risk of these behaviors decreasing as sleep duration approaches 8 or 9 h, before increasing as adolescents begin to oversleep [6]. As of 2019, 18.8% of high school students in the US reported considering suicide and 8.9% attempted suicide at least once [7]. Outside of sleep duration, adolescent suicidal ideation and attempts have been associated with factors including race/ethnicity [7], sexual orientation [8], and feelings of hopelessness [9].

Barring sleep disorders, inadequate adolescent sleep is primarily the result of two factors: late sleep initiation and early rise times [10,11]. Late sleep initiation stems from biological mechanisms surrounding the adolescent homeostatic sleep system and circadian rhythms [12,13] as well as behavioral factors such as homework, after school activities, and increased technology use [10,12,14,15]. Alternatively, the single most important predictor of early rise times is school start times. When adjusting for school start times, associations between early rise times and all of the aforementioned behaviors are attenuated [15]. Given the strong association between school start times and early rise times, a commonly proposed intervention for increasing the proportion of adolescents achieving adequate sleep has been to delay school start times.

While recent meta-analyses and systematic reviews show that the magnitude of effect varies across aggregated studies, they demonstrate a clear association between delayed school start times and longer sleep duration in adolescents [10,16,17]. Using data from the adolescent supplement of the US National Comorbidity Survey, Paksarian, et al. [18] found that for each 30 min delay in school start times prior to 8:01 a.m., sleep duration increased by over 11 min. The American Association of Pediatrics has suggested high school start times be delayed to 8:30 a.m. or later [14]. Schools with start times later than 8:30 a.m. have been shown to have as many as 60% of their students sleep 8 h or more on an average school night [19]. Additionally, students attending high schools starting after 8:30 a.m. had significantly later wake times [20], higher attendance [19,21], less tardiness [19], better academic performance [19], higher graduation rates [21], and lower rates of motor vehicle collisions [19,22] compared with students attending schools starting before 8:30 a.m.

There is also a clear association between delayed school start times and various depressive symptoms. Wahlstrom et al. reported a significantly lower proportion of students presenting with depressive mood at one high school following a delay in school start times from 7:15 a.m. to 8:40 a.m., though absolute differences were minimal [23]. Other researchers have observed decreases in student-reported depression and unhappiness ranging from just over 4.5% to greater than 20% following delays in start school start times [24,25,26,27]. Moreover, while a lower proportion of students reported depressive symptoms following a 25 min delay in a New England boarding school, students sleeping less than 8 h were more likely to report depressed mood compared with students sleeping 8 h or more regardless of their school start time [28], suggesting delayed school start times affect depressive symptoms by increasing sleep duration.

Though previous studies have identified an association between school start times and student sleep duration, estimates vary both for the proportion of students achieving an adequate amount of sleep and for the amount of additional sleep a delay may provide. Further, while the effects of school start times on depressive symptoms are well-researched, there is little to no research investigating the impact of delayed school start times on more serious adolescent mental health outcomes—namely, suicidal ideation and suicide attempts. These potential relationships are particularly important to elucidate as potential preventative measures in light of the increased prevalence of suicide attempts among adolescents in the United States from 2009 to 2019 [7].

To address these gaps in the literature, this study featured two specific aims. First, we aimed to quantify the association between school start times and sleep duration in high-school-aged adolescents. Our second aim was to investigate the associations between school start times and depressive mood, suicidal ideation, and attempted suicide.

## 2. Materials and Methods

### 2.1. Participants

Data for this study are from the 2019 Healthy Kids Colorado Survey (HKCS), a biennial, cross-sectional, and anonymous survey among public high school students in Colorado, United States. The HKCS uses a multi-stage cluster sampling design using a sampling frame of all public high schools in Colorado. The primary sampling unit is schools, with classrooms as the secondary units. Schools that participated could choose to administer the survey during a class period (e.g., homeroom) or subject (e.g., science) that was required for all students. Administration is consistent with the Centers for Disease Control and Prevention’s YRBS methodology [29]. Parents were informed in advance about the survey and, in most districts, could opt-out their students; a few districts required active parental consent. The sample consisted of 166 high schools (83% of selected schools) and 46,537 high school students (71% of sampled students), an overall response rate of 59%. The study was approved by the Colorado Multiple Institutional Review Board.

Prior to our analyses we examined student characteristics among participating schools (*n* = 166) and those that refused (*n* = 33) and found comparable distributions of gender and grade level. Although the distribution of race/ethnicity differed between responding and non-responding schools, there was no evidence of systematic differences. Among responding schools, about 5% more were in urban areas versus non-responding schools, and non-responding schools tended to have lower student enrollment.

### 2.2. Measures

#### 2.2.1. Sleep Duration

Students reported their hours of sleep in response to the following question, “On an average school night, how many hours of sleep do you get?” with the following response options: 4 or less hours, 5 h, 6 h, 7 h, 8 h, 9 h, or 10 or more hours. In addition to a 7-level sleep duration variable, responses were dichotomized into a 2-level sleep health variable: seven or less hours per night or eight or more hours, consistent with the American Association of Sleep Medicine’s adolescent sleep guidelines [2].

#### 2.2.2. Mental Health

Students were asked three questions regarding their mental health: if they felt sad or hopeless for two weeks or more in the past 12 months, considered suicide in the past 12 months, or attempted suicide in the past 12 months. Feelings of hopelessness and considering suicide were measured as yes/no, while suicidal attempt responses were dichotomized to none or once or more.

#### 2.2.3. Student Characteristics

Factors included in analyses were sex (male, female), grade level (9th, 10th, 11th, 12th), race/ethnicity (collapsed into non-Hispanic White, non-Hispanic Black, Hispanic, and any other race/multi-racial), sexual orientation (heterosexual vs. lesbian, gay, bisexual—LGB), and highest level of maternal education (less than college vs. some college or more).

#### 2.2.4. School Level Characteristics

School start times were determined through visiting school websites and reviewing publicly available information. If the information was unavailable online, a research assistant called the school and inquired about their start time. Consistent with the American Association of Pediatrics’ high school start time recommendations [14], schools starting at 8:30 a.m. or later was referred to as “late”, while schools starting before 8:30 a.m. were dichotomized as “very early” (<8:00 a.m.) and “early” (8:00–8:29 a.m.).

The National Center of Education Statistics’ (NCES) urban-centric locale codes were used to determine each school’s urbanicity classification. The 12 NCES levels were collapsed into three categories: urban (NCES locale codes: 11, 12, 13), rural (32, 33, 41, 42, 43), and suburban (21, 22, 23, 31) for ease of presentation. The proportion of students within a school qualifying for free and reduced lunch (FRL) was obtained from the Colorado Department of Education and was included in the analysis as a proxy for socioeconomic status.

### 2.3. Data Analysis

Student responses were weighted to state enrollment in public high schools in Colorado by sex, grade, and race/ethnicity to account for the sampling design, including clustering within schools and classrooms, school and classroom selection probability, nonresponse, and demographic discrepancies between the participating sample and state population.

We calculated weighted prevalence estimates and 95% confidence intervals (CIs) for student-level demographic factors including sex, grade, race/ethnicity, grade, sexual orientation, and mother’s education as well as adequate sleep and mental health behaviors, stratified by school start time. Descriptive statistics were calculated for school-level factors including urbanicity and FRL. Second order Rao–Scott chi-square tests were used to test for bivariate associations between school start times and each factor [30,31].

Weighted multivariate linear regression was used to estimate the impact on sleep duration per 15 min later school start time. Multivariate weighted logistic regression was used to estimate adjusted odds ratios (aORs) investigating the association between school start times and considering or attempting suicide. Demographic and school-level factors associated with school start times in the bivariate analysis (*p* ≤ 0.20) were included in linear and logistic regression models to control for potential confounding. In addition, feeling sad/hopeless was included in the logistic regression model as a proxy for depressed mood, while sexual orientation was included on theoretical grounds due to its association with suicide-related behaviors [8]. Statistical analyses were performed using complex survey procedures in SAS 9.4 (SAS Institute Inc, Cary, NC, USA) with an alpha level of 0.05.

## 3. Results

### 3.1. Bivariate Analysis

Eighty-six high schools (51.8%) were classified as having very early start times, ranging from 7:10 a.m. to just before 8:00 a.m., 60 (36.1%) had start times from 8:00 a.m. to 8:29 a.m., and 20 (12.1%) had late start times ranging from 8:30 a.m. to as late as 9:00 a.m. There were no significant differences in school start time by student sex, grade, or sexual orientation (Table 1), with approximately half of the students being male (51.1%), a quarter in each grade, and 88% heterosexual. Race/ethnicity was not associated with school start time, though schools with late start times had a higher proportion of non-Hispanic White students compared to schools with early or very early start times. Feelings of sadness/hopelessness were also not associated with school start time.

Students attending schools with a late start time were more likely to have mothers who had at least some college education. School urbanicity was significantly associated with school start time. Relative to the schools with earlier start times, schools with late start times were more likely to be in urban areas (50.0%) versus suburban (35.0%) or rural (15.0%). Schools with late start times had a significantly lower proportion of students qualifying for FRL.

Student reported hours of sleep was significantly associated with school start time; 23.2% (95% CI: 22.0–24.4) of students in schools with very early start times reported receiving 8 h of sleep or more on an average school night compared to 32.2% (95% CI: 29.5–35.0) of students in schools with late start times (Table 2). Significantly more students reported attempting suicide both in schools with very early start times (8.0%, 95% CI: 7.5–8.5) and early start times (7.9%, 95% CI: 7.0–8.7) compared to those with late start times (6.1%, 95% CI: 5.5–6.8%; *p* = 0.03), though no significant association was observed for suicidal ideation.

When examining the prevalence of suicidal ideation by mental health and demographic factors, little difference was observed across school urbanicity or maternal education, at approximately 17% (Table 3). A higher prevalence of suicidal ideation was observed in other/multi-racial students (20.6%), students feeling sad or hopeless (43.3%), and LGB students (42.0%). Similarly, suicide attempts were more prevalent in students within racial/ethnic minorities (8.6% to 10.1%), those with feelings of sadness or hopelessness (19.2%), and LGB students (20.7%) as well as those with lower maternal education (10.5%).

### 3.2. Multivariate Analysis

In an adjusted linear regression of school start time on student reported hours of sleep, each 15 min later school start time was associated with 4.6 (95% CI: 3.4–5.9) more minutes of sleep on an average school night, controlling for student race/ethnicity, maternal education, school urbanicity, and FRL status (Supplementary Table S1).

Table 3 presents adjusted models of student mental health outcomes regressed on individual and school level factors. Relative to students attending schools with late start times, students at schools with very early start times had 1.10 (95% CI: 0.95–1.27) times the odds of attempting suicide, while those attending schools with early start times had 1.11 (95% CI: 0.98–1.27) times the odds, controlling for feeling sad/hopeless, race/ethnicity, sexual orientation, urbanicity, maternal education, and the proportion of students utilizing FRL. There was no significant association between school start times and considering suicide (Table 3).

Feeling sad or hopeless for two weeks or more had the highest magnitude of association for both considering and attempting suicide. Relative to White non-Hispanic students, those of all other race/ethnic groups had higher odds of attempting suicide at least one time. Additionally, LGB students had higher odds of both behaviors compared with heterosexual students. Students’ whose mother’s education level was some college or above were significantly less likely to have attempted suicide than those with mother’s education of less than college. Lastly, the geographic setting of the school was not a significant factor for either outcome.

## 4. Discussion

This study used a large sample of high schools from a population-based surveillance system to investigate associations between high school start times and various student- and school-level factors. We found that student maternal education, school urbanicity, and school FRL status were associated with school start times. Students attending schools with late start times were more likely to have mothers with at least some college education and to attend schools in urban locations, with lower proportions of students receiving FRL compared to students attending schools with earlier start times. Further, students were more likely to sleep 8 h or more when attending a school with a late start time. We observed an association between school start time and sleep duration, with each 15 min later school start time corresponding to 4.6 more minutes of sleep on an average school night. School start times were marginally associated with suicide attempts but not suicidal ideation, within our study population after adjusting for student- and school-level factors.

Our findings regarding the association between later school start times and longer sleep duration are consistent with the positive aggregated associations observed in recent meta-analyses on the subject [16,17]. Approximately 32% of students attending schools with late start times slept 8 h or more on an average school night. This proportion is similar to the United States Government’s Healthy People 2020 sleep duration target for high-school-aged adolescents [32], while the 23% of students sleeping 8 h or more attending schools with very early start times are similar to the 2019 national average as measured by the YRBS [3]. The significant difference between these groups is consistent with the delay of school start times functioning as an effective intervention for increasing the proportion of students sleeping 8 h or more to target levels.

While we observed a positive association between later school start times and sleep duration, the proportion of students achieving 8 h or more of sleep on an average school night within schools with late start times was lower than that of other recent studies. Wahlstrom, et al. [19] saw over 55% of students sleeping 8 h or more in districts with start times later than 8:30 a.m., similar to Owens, et al. [24]. That said, other studies found sleep duration to be between 4.8 to 6.2 min longer per 15 min delay in school start time [18,20,33], comparable with our estimate after adjusting for similar covariates. Given that this study was conducted across a large sample of schools in one state compared to the nationally representative estimates presented in the aforementioned studies, these differences raise questions as to why later school start times seem to have less of an impact on the proportion of students sleeping 8 h or more in Colorado than as observed elsewhere.

Schools with earlier start times had a higher proportion of racial/ethnic minority students relative to schools with late starts. Further, students belonging to any racial/ethnic minority had higher odds of attempting suicide compared to non-Hispanic White students. Given that students of racial/ethnic minorities appear less likely than White students to achieve adequate sleep [34], and given the variety of negative health outcomes associated with inadequate sleep [5], including suicidal ideation and attempts [6], it is possible that early school start times contribute to the development and maintenance of health disparities within minority students through their impact on inadequate sleep.

Further, the differences in both race/ethnicity and maternal education between students attending high schools with a late start time compared to schools with earlier start times indicate potential individual-level socioeconomic influences on school start time policies. When these differences are taken in conjunction with the lower proportion of students receiving FRL in schools with late start times, a measure of school-level socioeconomic status, lower socioeconomic status appears to be associated with earlier school start times.

In a survey administered to 345 high schools across the United States, Wolfson and Carskadon [35] observed later school start times in schools with lower school-level socioeconomic status, contrasting these results. As Colorado has one of the lowest poverty rates within the United States, individual- and school-level socioeconomic status could be operating differently with regard to school start time policy as compared to the national level [36,37]. Given the difference between these results, we believe it is important to further investigate and quantify socioeconomic disparities in the implementation of school start time delays.

Urban schools were more likely to have a late start time compared to rural and suburban schools, similar to results first reported by Paksarian, et al. [18]. The authors posit school transportation as a potential reason for this discrepancy, with bussing differences impacting urban/rural start times [18,26]. Rural schools have been found to have longer bus routes compared to suburban schools and are more likely to require students from different school levels (elementary/middle/high) to share busses, likely impacting school start times [38].

We found no significant difference in the proportion of students feeling hopeless or sad when comparing schools with earlier start times to those with late start times, contrasting the results of several other studies [23,24,25,26,27,28]. It is possible that while late school start times were associated with longer sleep duration within our study population, the students were not getting a sufficient amount of additional sleep to have a discernable impact on their depressive mood.

This study was the first to investigate the associations between school start times, sleep duration, and both suicidal ideation and attempts among a large sample of high-school-aged adolescents. Feeling hopeless or sad was the strongest predictor of both suicidal ideation and attempts within our study population, consistent with studies using similar survey methodology [39]. Relative to late start times, early and very early school start times saw a marginal, though not statistically significant, association with attempted suicide: 10% and 11% higher odds, respectively, controlling for all other individual and school-level factors. Further research into the interplay between school start times, sleep, and mental health outcomes is necessary to verify this relationship.

Our study is not without limitations. We utilized data from a cross-sectional study, limiting the ability for causal inference. The data were weighted to be representative of adolescents attending high school in the state of Colorado. As mentioned previously, Colorado has one of the lowest poverty rates in the United States (9.6% vs. 11.8%) [36,37] and has a 7.5% higher proportion of Non-Hispanic Whites than the United States as a whole [37]. These factors may impact this study’s generalizability.

The overall response rate for the HKCS was 59%. If there were systematic differences between non-respondents and those completing the survey, other than demographic factors considered in weighting, it is possible selection bias influenced the observed results. Social desirability bias should also be considered given the sensitive nature of the questions regarding mental health outcomes and students being administered the survey with their peers.

Sleep duration was recorded in hourly increments as a categorical variable on the HKCS. However, our estimate of the linear relationship of sleep duration on school start time was presented in minutes to facilitate comparisons with prior studies that measured sleep duration in minutes. The differences in measurements between this and other studies should be taken into account when interpreting this relationship. Lastly, students were only questioned on their weeknight sleeping habits, so these results are not applicable to associations between school start times and weekend sleep duration.

Our study also features several strengths. By collecting data from over 46,000 students in 166 schools, this is one of the largest single studies investigating the associations between delayed school start times and sleep duration in adolescents. Data collection was consistent with the Centers for Disease Control and Prevention’s YRBS methodology and utilized the standard YRBS questions for the variables of interest. These methodological considerations should facilitate comparisons between our study and other state- or city-level YRBS-based surveys where school start time data are available. Finally, to our knowledge, this is the first study investigating the associations between school start times and outcomes surrounding suicidal ideation and attempts.

This study highlights avenues for additional research. Longitudinal studies should be conducted to further investigate and verify associations between school start times and attempted suicide. These studies should incorporate an expanded analysis of potential covariates or feature a qualitative component to identify the underlying mechanism of this association. Additionally, similar studies should be conducted in other states to uncover why later school start times seemed to have less of an impact on the proportion of students sleeping 8 h or more in Colorado compared with other national-level studies. Lastly, additional research is necessary to describe potential socioeconomic and regional disparities in the implementation of school start time delays.

## 5. Conclusions

These data further support the association between later school start times and longer sleep duration in high-school-aged adolescents. While at an individual level, changes in sleep duration may seem minimal, they have significant impacts on the proportion of students sleeping 8 h or more. That said, later school start times were more prevalent among students with a higher socioeconomic status and attending urban schools. These factors should be considered for ensuring equitable implementation of school start time delays. Further, school start times were marginally associated with suicidal attempts, controlling for various student- and school-level factors. Though not an easy change, these results support delaying school start times as an effective intervention for increasing students’ sleep duration. Additionally, student mental health outcomes should continue to be investigated when assessing the impact of delayed start times.

## Figures and Tables

**Table 1:** Individual and school-level demographic characteristics.

Level	Factor	Overall, % (95% CI)	Schools with Very Early Start Times, % (95% CI)	Schools with Early Start Times, % (95% CI)	Schools with Late Start Times, % (95% CI)	*p*-Value
*Students*	*Total Responses ^a^*	46,537	21,236 (53.2%)	18,754 (28.2%)	6547 (18.5%)	
	*Sex*					0.92
	Female	48.9 (48.3, 49.6)	48.9 (47.9, 49.8)	48.8 (47.8, 49.8)	49.3(47.7, 50.8)	
	Male	51.1 (50.4, 51.7)	51.1 (50.2, 52.1)	51.2 (50.2, 52.2)	50.7 (49.2, 52.3)	
	*Grade*					0.53
	9th	26.8 (25.9, 27.7)	27.8 (26.7, 28.9)	26.2 (24.4, 28.1)	24.9 (23.1, 26.8)	
	10th	25.8 (24.7, 26.9)	25.4 (24.1, 26.8)	26.8 (24.2, 29.3)	25.4 (23.9, 27.0)	
	11th	24.1 (23.1, 25.0)	23.7 (22.3, 25.0)	24.5 (23.0, 26.0)	24.5 (22.6, 26.4)	
	12th	23.3 (22.4, 24.2)	23.1 (21.9, 24.3)	22.6 (21.0, 24.1)	25.1 (23.0, 27.1)	
	*Race/Ethnicity*					0.20
	Non-Hispanic White	55.1 (51.7, 58.4)	55.0 (49.0, 61.1)	49.7 (44.4, 55.0)	63.2 (57.2, 69.2)	
	Non-Hispanic Black	4.3 (3.5, 5.2)	4.7 (3.2, 6.3)	5.5 (3.7, 7.4)	1.4 (1.1, 1.6)	
	Hispanic	32.2 (29.0, 35.4)	31.7 (26.0, 37.4)	36.0 (30.7, 41.2)	27.9 (21.8, 34.1)	
	Other ^b^/Multi-Racial	8.4 (7.7, 9.0)	8.5 (7.6, 9.4)	8.7 (7.3, 10.2)	7.5 (6.3, 8.7)	
	*Sexual Orientation*					0.99
	Heterosexual	87.8 (87.1, 88.5)	87.8 (86.7, 88.9)	87.7 (86.4, 89.0)	87.9 (86.8, 88.9)	
	LGB	12.2 (11.5, 12.9)	12.2 (11.1, 13.3)	12.3 (11.0, 13.6)	12.1 (11.1, 13.2)	
	*Mental Health*					0.26
	Felt sad or hopeless	34.7 (33.9, 35.5)	35.3 (34.0, 36.7)	34.4 (33.2, 35.6)	33.2 (31.9, 34.4)	
	*Mother’s Education*					0.03
	Less than college	31.6 (28.9, 34.2)	31.7 (27.2, 36.2)	37.1 (32.2, 42.1)	22.9 (18.4, 27.3)	
	Some college or above	68.4 (65.8, 71.1)	68.3 (63.8, 72.8)	62.9 (57.9, 67.8)	77.1 (72.7, 81.6)	
*Schools*	*Total Participating*	166	86 (51.8%)	60 (36.1%)	20 (12.1%)	
	*Urbanicity*					<0.01
	Urban	41 (24.7)	20 (23.3)	11 (18.3)	10 (50.0)	
	Suburban	45 (27.1)	28 (32.6)	10 (16.7)	7 (35.0)	
	Rural	80 (48.2)	38 (44.2)	39 (65.0)	3 (15.0)	
	*Free and reduced lunch ^c^*	38.8 (20.7)	40.7 (21.8)	39.7 (20.0)	28.2 (15.2)	0.05

This table collects the prevalence of various student- and school-level factors, overall and stratified by school start time. Student-level factors are weighted to reflect Colorado’s high school population. School-level factors are presented as the unweighted number of schools agreeing to participate in the HKCS (unweighted percentage). The presented *p*-values test for bivariate associations and are estimated based on the second order Rao–Scott chi-square test. ^a^ Unweighted number of students completing the HKCS (weighted percentage); ^b^ other/multi-racial: Asian, American Indian/Alaska Native, Native Hawaiian/Pacific Islanders; ^c^ unweighted mean percentage (standard deviation) of students enrolled in FRL. Abbreviations: CI, confidence interval; LGB, Lesbian, Gay, Bisexual.

**Table 2:** Prevalence of sleep and mental health outcomes by school start time.

Level	Factor	Overall, % (95% CI)	Schools with Very Early Start Times, % (95% CI)	Schools with Early Start Times, % (95% CI)	Schools with Late Start Times, % (95% CI)	*p*-Value
*Students*	*Total Responses*	46,537	21,236 (53.2%)	18,754 (28.2%)	6547 (18.5%)	
	*Sleep Duration*					<0.01
	4 h or less	8.9 (8.5, 9.3)	10.4 (9.9, 11.0)	7.8 (7.2, 8.3)	6.1 (5.0, 7.1)	
	5 h	12.3 (11.8, 12.8)	13.8 (13.1, 14.6)	11.4 (10.6, 12.2)	9.4 (8.2, 10.6)	
	6 h	23.3 (22.7, 23.9)	24.7 (23.8, 25.6)	22.7 (22.0, 23.5)	20.0 (18.2, 21.7)	
	7 h	29.5 (29.0, 30.1)	27.8 (27.0, 28.7)	31.0 (29.8, 32.1)	32.3 (31.1, 33.5)	
	8 h	19.9 (19.3, 20.5)	17.8 (16.9, 18.7)	20.7 (20.0, 21.4)	24.4 (22.5, 26.4)	
	9 h	4.6 (4.3, 4.8)	3.7 (3.3, 4.1)	4.8 (4.3, 5.2)	6.7 (5.8, 7.6)	
	10 h or more	1.6 (1.4, 1.7)	1.7 (1.5, 1.9)	1.7 (1.5, 1.9)	1.1 (0.8, 1.4)	
	*Sleep Health*					
	8 h or more	26.0 (25.2, 26.8)	23.2 (22.0, 24.4)	27.2 (26.2, 28.2)	32.2 (29.5, 35.0)	<0.01
	*Mental Health*					
	Considered suicide, past 12 months	17.5 (17.0, 17.9)	17.8 (17.0, 18.5)	17.2 (16.4, 17.9)	17.0 (16.0, 18.0)	0.56
	Attempted suicide, past 12 months	7.6 (7.2, 8.0)	8.0 (7.5, 8.5)	7.9 (7.0, 8.7)	6.1 (5.5, 6.8)	0.03

This table collects the prevalence of sleep duration, sleep health, suicidal ideation, and suicide attempts, both overall and stratified by school start time. The presented *p*-values test for bivariate associations and are estimated based on the second order Rao–Scott chi-square test. Abbreviations: CI, confidence interval; h, hours.

**Table 3:** Association between suicide-related behaviors and school start time, adjusting for mental health and demographic factors.

Factor	Consider Suicide			Attempt Suicide		
	Prevalence (%)	*n* ^c^	aOR (95% CI)	Prevalence (%)	*n* ^c^	aOR (95% CI)
*School start time*						
Late: ≥8:30 a.m.	17.0	1147	1.00	6.1	463	1.00
Early: 8:00–8:29 a.m.	17.2	3133	1.07 (0.97, 1.17)	7.9	1469	1.11 (0.98, 1.27)
Very Early: <8:00 a.m.	17.8	3702	1.06 (0.95, 1.18)	8.0	1742	1.10 (0.95, 1.27)
*Feel sad or hopeless*						
Never felt sad/hopeless more than 2 weeks	3.9	1109	1.00	1.5	441	1.00
Ever felt sad/hopeless more than 2 weeks	43.3	6857	**16.38 (14.63, 18.33)**	19.2	3200	**13.77 (11.74, 16.16)**
*Race/Ethnicity*						
Non-Hispanic White	17.3	4102	1.00	6.1	1522	1.00
Non-Hispanic Black	16.1	184	1.02 (0.77, 1.35)	8.6	112	**1.53 (1.10, 2.12)**
Hispanic	17.0	2653	**0.88 (0.80, 0.96)**	9.4	1510	**1.30 (1.15, 1.48)**
Other/Multi-Racial ^a^	20.6	839	**1.19 (1.04, 1.37)**	10.1	422	**1.64 (1.39, 1.93)**
*Sexual orientation*						
Heterosexual	13.4	5052	1.00	5.4	2179	1.00
LGB	42.0	2139	**2.54 (2.29, 2.82)**	20.7	1087	**2.42 (2.13, 2.74)**
*Urbanicity*						
Urban	17.3	2270	1.00	7.6	1054	1.00
Suburban	17.7	2674	1.01 (0.92, 1.11)	7.6	1160	**1.16 (1.01, 1.32)**
Rural	17.2	3038	1.03 (0.93, 1.14)	7.7	1460	1.10 (0.98, 1.23)
*Mother’s education*						
Less than college	20.0	2710	1.00	10.5	1430	1.00
Some college or above	17.0	4363	1.00 (0.92, 1.09)	6.0	1679	**0.80 (0.71, 0.89)**
*Free and reduced lunch ^b^*	-	-	0.99 (0.98, 1.00)	-	-	**1.03 (1.02, 1.04)**

This table collects adjusted odds ratios (aORs) generated from the survey-weighted multivariate logistic regression of suicidal ideation and suicide attempts on selected student- and school-level factors. Each aOR is presented adjusting for all other factors in the model, with those reaching statistical significance at α = 0.05 bolded. ^a^ Other/multi-racial includes Asian, American Indian/Alaska Native, Native Hawaiian or other Pacific Islanders, or multi-racial; ^b^ the odds ratio for every 5% increase in the proportion of students qualifying for free and reduced lunch programs; ^c^ the unweighted number of participants. Abbreviations: CI, confidence interval; LGB, Lesbian, Gay, Bisexual.

## Data Availability

The data presented in this study are available on request from the corresponding author. The data are not publicly available in order to protect the confidentiality of the participating schools.

## Supplementary Materials

The following are available online at https://www.mdpi.com/article/10.3390/ijerph18115708/s1, Supplementary Table S1: Multivariate linear regression of school start time on sleeping hours.
