# Supplementary material for: The Relationship of School Start Times, Sleep Duration and Mental Health among a Representative Sample of High School Students in Colorado, 2019
Source: Int J Environ Res Public Health. 2021 May 26;18(11):5708. doi: 10.3390/ijerph18115708 (PMC8198323; doi:10.3390/ijerph18115708)
Supplement: Supplementary file 1 [file ijerph-18-05708-s001.zip › ijerph-1209390-supplementary.pdf]

Table S1. Multivariate linear regression of school start time on sleeping hours.

Predictor Variables	Unit	Coefficient	95% CI	*p-Value*
School Start Time	15 minutes	4.6	3.4, 5.9	<0.0001
Race/Ethnicity	Category			
Non-Hispanic White		0	-	-
Non-Hispanic Black		-11.8	-19.0, -4.6	0.0014
Hispanic		-1.5	-5.8, 2.8	0.4892
Other/Multi-Racial		-11.6	-16.1, -7.1	<0.0001
Mother's Education	Dummy			
Less than college		0	-	-
Some college or above		9.5	6.2, 12.7	<0.0001
Urbanicity	Category			
Urban		0	-	-
Suburban		-3.9	-10.4, 2.6	0.2371
Rural		7.2	3.1, 11.3	0.0008
Free and reduced lunch	5%	0.41	-1.0, 0.2	0.1712
